# The effect of contamination disgust on Covid-19-related anxiety

**DOI:** 10.1192/j.eurpsy.2022.211

**Published:** 2022-09-01

**Authors:** G. Santarelli, M. Innocenti, V. Faggi, F. Galassi, G. Castellini, V. Ricca

**Affiliations:** 1 University of Florence, Human Health Sciences, Firenze, Italy; 2 University of Florence, Human Health Sciences, firenze, Italy; 3 University of Florence, Department Of Health Sciences, Florence, Italy

**Keywords:** Covid-19, contamination disgust, Anxiety, Covid-19-related anxiety

## Abstract

**Introduction:**

It is proven that high levels of disgust contribute to implementing protective behaviors. Investigators also discovered that the emotion of disgust plays a central role in determining anxiety related to the contraction of COVID-19. Few data are available about the role of the contamination disgust, a specific disgust domain, in this relationship.

**Objectives:**

The effect of contamination disgust on COVID-19-related anxiety was investigated.

**Methods:**

295 healthy subjects were enrolled through an online survey. They completed Disgust Scale-Revised (DS-R) and were asked to estimate their levels of Covid-19-related anxiety in 12 proposed situations. A total score was then calculated. An ANOVA model having Covid-19-related anxiety total score as dependent variable, and DS-R contamination disgust, age, and sex as predictors was estimated.

**Results:**

The overall model was significant (F(3,291)=6.402, p<0.001) and explained 6.2% of total Covid-19 anxiety variance (R^2^=0.062). The effect of DS-R contamination disgust on Covid-19-related anxiety was positive, significant (B=0.974, t(291)=3.227, p=0.001) and explained 3.5% of Covid-19-related anxiety variance (partial η^2^=0.035). A significant effect of sex was detected (F(1,291)=4.919, p=0.027), with females having higher Covid-19-related anxiety than males, while no effect was detected for age (B=-0.024, t(291)=-0.884, p=0.377).

**Figure 1:**
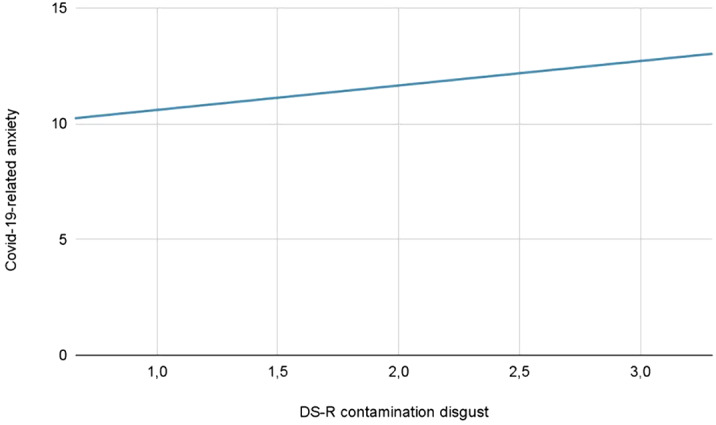

**Conclusions:**

The presented data provide preliminary evidence for an effect of contamination disgust on Covid-19-related anxiety.

**Disclosure:**

No significant relationships.

